# Development and evaluation of a web-based breast cancer cultural competency course for primary healthcare providers

**DOI:** 10.1186/1472-6920-11-59

**Published:** 2011-08-15

**Authors:** Richard C Palmer, Raquel Samson, Maria Triantis, Irene D Mullan

**Affiliations:** 1 Robert Stempel College of Public Health and Social Work. 11200 SW 8th Street, Miami, FL, 33199, USA; 2 8757 Georgia Avenue, 10th Floor, Silver Spring, Maryland 20910, USA; 3 Cancer and Tobacco Initiatives, Montgomery County Department of Health and Human Services. 1335 Piccard Drive, Rockville, MD 20850, USA

## Abstract

**Background:**

To develop and evaluate a continuing medical education (CME) course aimed at improving healthcare provider knowledge about breast cancer health disparities and the importance of cross-cultural communication in provider-patient interactions about breast cancer screening.

**Methods:**

An interactive web-based CME course was developed and contained information about breast cancer disparities, the role of culture in healthcare decision making, and demonstrated a model of cross-cultural communication. A single group pre-/post-test design was used to assess knowledge changes. Data on user satisfaction was also collected.

**Results:**

In all, 132 participants registered for the CME with 103 completing both assessments. Differences between pre-/post-test show a significant increase in knowledge (70% vs. 94%; p < .001). Ninety-five percent of participants agreed that the web based training was an appropriate tool to train healthcare providers about cultural competency and health disparities.

**Conclusion:**

There was an overall high level of satisfaction among all users. Users felt that learning objectives were met and the web-based format was appropriate and easy to use and suggests that web-based CME formats are an appropriate tool to teach cultural competency skills. However, more information is needed to understand how the CME impacted practice behaviors.

## Background

Mammography screening has been found to be effective in reducing deaths from breast cancer [1], yet underserved woman are more likely to die from this disease [2,3]. Underserved women are less likely to be screened, which results in later stage diagnosis and decreased survival rates [1]. Imperative to decreasing and eliminating breast cancer disparities is ensuring timely and regular mammography screening for underserved women to ensure early detection [1-3].

Breast cancer disparities research has attributed factors such as access to care, socioeconomic status, genetics, and tumor biology as possible causes of this inequity [1,4]. However, it is not well known how these factors, singularly or in combination, contribute to the disparity seen for breast cancer. An additional factor, culture, has been hypothesized as a possible mediator of health disparities in general and for breast cancer [5]. Although the exact contribution that culture attributes to breast cancer disparities is not well known, observational studies have identified that cultural beliefs and attitudes can influence the decision to seek breast care and screening [5,6]. As a framework, culture affect's a patient's perception of disease and care seeking, and influences how a patient communicates with healthcare providers and navigates healthcare settings [7-10]. For breast cancer, culture has been shown to influence breast cancer risk perception, knowledge, beliefs about cure, as well as reinforcing health and illness behaviors surrounding prevention and treatment [5]. Although identified as factor that contributes to breast cancer disparities, culture is very complex with many different subcultural variations within groups, which contributes to a mosaic of different norms, attitudes, and behavioral beliefs about breast cancer [5].

In the United States (U.S.) there has been increased recognition of the importance of culture and the role of cultural competence within healthcare settings [11,12]. As a result, healthcare settings have adapted to provide culturally and linguistically appropriate services to socio-cultural diverse individuals to ensure better access and improved health care [13,14]. However, as part of the educational process on cultural competency, there has been a tendency to homogenize the role of culture in health, possibly perpetuating culturally-based stereotypes in healthcare delivery [15]. Healthcare providers should recognize that there are unique differences within cultural and subcultural groups and can affect how patients view illness. Therefore, central to cultural competence in the healthcare environment is the ability to acknowledge different reference points in physician-patient interactions, the ability to understand patients' culturally rooted health beliefs and practices, and to negotiate treatment that aligns with patients' cultural value systems [16,17]. For breast cancer screening, providers must recognize that women of different cultural groups have different beliefs and values that can influence adherence with a screening recommendation [11,17].

By 2050 approximately half of the U.S. population will be comprised of racial/ethnic minority groups with many speaking a primary language other than English [18,19]. As a result, inability to effectively communicate with a healthcare provider may occur and will create access barriers [12,20]. Lack of a common language between client and provider can result in diagnostic errors and inappropriate treatment [21]. Further, lack of cultural awareness between patient and healthcare provider can lead to mistrust, perceived discrimination, and decreased likelihood of adherence [22]. Conversely, culturally competent care has the capacity to improve access to health care services, minimize medical errors, and increase the rate of use of preventive services [23,24]. Additionally, cultural competency may improve patient adherence and satisfaction, decrease financial costs and ultimately help in the elimination of health care disparities [25,26].

The purpose of the study was to develop and evaluate a web-based continuing education (CME) course to educate healthcare providers about breast cancer health disparities, the role culture has on influencing patient decision making, and to provide a cross-cultural framework for healthcare providers to use that could enable them to better discuss breast cancer screening with socio-cultural diverse patients. This CME was part of a multi-component program aimed at identifying how community-based health centers (Montgomery Cares) located in Montgomery County, Maryland coordinate care regarding breast health and mammography. Data obtained by medical record abstraction from a needs assessment conducted in Montgomery Cares community health centers revealed that mammography screening rates were low (12%) and that healthcare providers did not regularly recommend mammography screening to their patients [27]. Therefore, we developed the CME course with the following aims: 1) to increase awareness about breast cancer morbidity and mortality; 2) to increase healthcare providers understanding of factors that influence decision to undergo breast cancer screening; 3) to elucidate how culture and cultural competence can influence breast cancer services; and 4) to develop skills that healthcare providers can use to help recommend breast cancer screening to women of diverse backgrounds.

## Methods

### Program Development

The interactive web-based CME was developed to include video, graphics, and text to educate healthcare providers about breast cancer, the role of culture and its impact on health decision making, and demonstrated an effective strategy for cross-cultural health communication with patients. A web-based CME platform was chosen since formative work conducted with healthcare providers who primarily work in community health centers in Maryland, our primary target audience, rated web-based CME instruction as their preferred medium for receiving continuing education. Once the web-based user interface was identified, a multimedia company experienced in developing web-based instructional programs was hired to develop the user interface, graphics, and video.

To create the CME, the study team initially drafted a story board which outlined CME content in four modules during the summer of 2007. The story board was then shown to primary care providers, breast cancer oncologists, and experts in healthcare cultural competence for their input and feedback. Once feedback was received, the study team modified the story board to address comments made by the reviewers. Next, a script was developed by the study team with corresponding text and graphics. These materials were shown to the reviewers for their input and content was modified to ensure scientific accuracy and clarity. Once the script and accompanying text and graphics were finalized, the multimedia company filmed the narration and vignettes, created a beta version of the web-based CME, and developed a corresponding database to collect user demographics, CME test responses, and process measures. Beta testing of the web-based CME was conducted by the study team and ten potential end users which resulted in modifications that made the CME easier to use. The Maryland State Medical Society, MedChi, approved content for 1 CME credit hour.

### Program Content

The content of the CME was organized around four modules: 1) Breast Cancer Epidemiology; 2) Breast Cancer Screening; 3) Culture and Cultural Competence; 4) Cross-Cultural Health Communication. Modules were designed for primary care providers and were approximately 15 minutes in length. After registration, the user was directed to the CME introduction where the CME narrator welcomed the participant and provided an overview of the course and the four sections. After watching the introduction, participants were prompted to begin module 1.

#### Module 1: Breast Cancer Epidemiology

**Figure 1:**
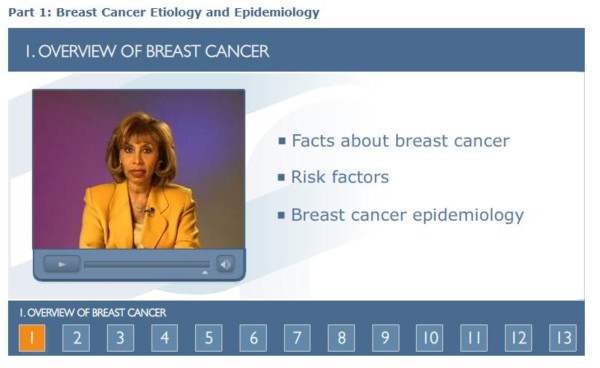
**CME Part 1 Introduction**.

Module 1 provided the learner with an overview of breast cancer etiology and epidemiology (Figure 1). The module provided an overview of the most common types of breast cancer and presented and epidemiological overview of breast cancer in the United States based on SEER data. Breast cancer disparities in incidence, staging, and survival were also covered. Additionally, an overview of the risk factors that have been associated with increased breast cancer risk were presented.

#### Module 2: Breast Cancer Screening

Module 2 reviewed guidelines for mammography screening and clinical breast exam. The module presented recommendations from U.S. Guide for Preventive Services, the American Cancer Society, and National Cancer Institute. The module also addressed differences in the recommendations of these agencies. Further, the module also provided mammography screening prevalence rates by age and race from the National Health Interview Survey and Behavioral Risk Factor Surveillance System data.

#### Module 3: Culture and Cultural Competence

**Figure 2:**
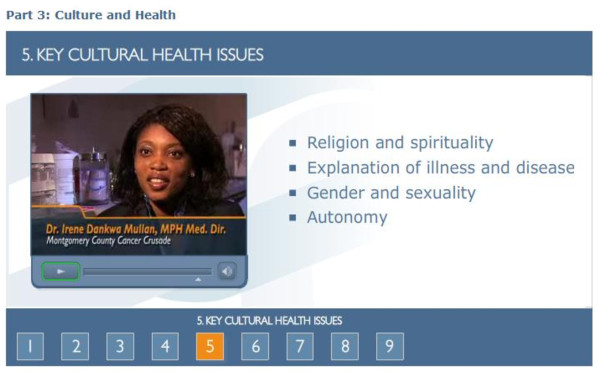
**Personal Testimonial from Healthcare Practitioner**.

In this module, the learner was presented with an overview of what culture is and how culture can influence breast cancer screening decisions of women. Specifically, this module explored the role of culture and health and presented evidence that the experience of illness and perception of disease varies by culture and that culture influences help seeking and health care utilization. Additionally, the module discusses the role of cultural competence in U.S. healthcare systems, discussed the National Standards on Culturally and Linguistically Appropriate Services (CLAS) in Health Care, and highlighted the benefits of cultural competent healthcare. Intertwined throughout this module were video vignettes of healthcare providers discussing their experiences with recommending breast cancer screening and clinical breast examination to women of diverse cultural backgrounds (Figure 2). Further, vignettes of patients were also presented that highlighted how patients' cultural backgrounds influenced their health care decision making about breast cancer screening.

#### Module 4: Cross-Cultural Health Communication

**Figure 3:**
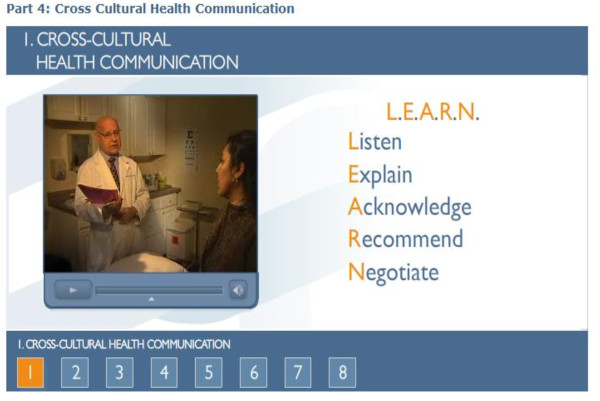
**LEARN Vignette**.

In the final module, we provided a communication framework, the LEARN mnemonic (Listen sympathetically to the patient's perception of the problem, Explain your perceptions of the problem, Acknowledge and discuss differences and similarities, Recommend a treatment plan, Negotiate agreement [28], and demonstrated how LEARN could be used during a patient interview. To demonstrate LEARN, a vignette that showed a physician -patient interaction about breast cancer screening was included in the module (Figure 3). The module then demonstrated how the physician used the components of LEARN to plan a breast cancer screening strategy that was acceptable to the patient.

### CME Evaluation

A single group pre-test/post-test design was used to evaluate the CME. Before participants commenced each module, a series of pretest questions reflecting the main learning objectives of each module were asked to establish a baseline. In total, there were 10 pretest questions. Immediately after completing the fourth module, the same 10 questions were then asked during the post-test. When a participant answered 70% of the post-test questions correctly, they received a certificate of completion for passing the CME course. Additionally, participants were asked to complete twelve Likert scale (strongly disagree to strongly agree) process evaluation questions to rate their satisfaction with the course and content.

### Data Analysis

Descriptive frequencies and percentages were calculated to characterize CME participants and their responses to process evaluation items. Individuals who audited the course and who did not have post-test data were excluded from analysis (n = 29). To assess if there were changes between pre-test and post-test among individual items, McNemar's chi square test was used due to the nonparametric nature of the data. Results were considered significant when *P *< .05. Data were analyzed using SPSS v17.0 [29].

## Results

In all, 132 participants registered for the CME with 103 completing both the pre-test and post-test (Table 1). Participants tended to be female (89.9%) and non-Hispanic white (58%). A little more than half of participants who registered were between the ages of 35-55 years. The majority of participants who completed the CME course were registered nurses (32%), followed by physicians (20.4%) and then nurse practitioners (14.4%). Of individuals who practiced, 96% reported practicing in an urban setting.

**Table 1:** Sociodemographic Characteristics of CME Participants (N = 132)*

	N	%
**Gender**		

Male	16	11.1

Female	116	88.9

**Race/Ethnicity**		

White	77	58.3

African American/Black	16	12.2

Asian/Pacific Islander	12	9.0

Hispanic/Latino	8	6.0

American Indian/Alaska Native	1	0.8

Other	8	6.0

**Age**		

< 35	23	17.4

35 - 54	71	54.0

≥55	35	27.0

**Profession**		

Nurse	43	32.3

Nurse Practitioner	19	14.4

Physician	27	20.4

Other Healthcare	25	18.9

**Practice Specialty**		

Family Practice	28	21.2

Internal Medicine	5	3.7

Obstetrics and Gynecology	7	5.3

Oncology	16	12.2

Surgery	5	3.7

Other specialty	12	9.0

Not in practice	17	12.8

**Percent Minority Patients Served in Practice**		

≤ 20	24	18.2

21-40%	21	15.9

41-60%	16	12.1

≥61	33	25.0

**Practice Setting**		

Community Health Center	22	16.7

Hospital	24	18.2

Clinic	19	14.4

Other	17	12.9

*Totals may not add to 100% due to missing data.

Only 45% of participants answered 70% of the pre-test questions correctly as compared to nearly 94% at post-test (p < .001). Significant pre-post changes were obtained for six out of the ten CME test questions (Table 2). An additional question about the causes of breast cancer disparities approached significance (p = .06), while a question about the role of culture in health showed no change between pre- and post-test. Of the questions that did change between pre- and post-test, participants were able to correctly identify the most commonly diagnosed breast cancer in women in the United States (p = .001), percent of breast cancers in women diagnosed at a localized stage (p < .001), the racial/ethnic group least likely to participate in routine mammography screening (p < .001), identify that the rate of mammography screening is decreasing (p < .001), and understood the major components of the LEARN model (p < .001).

**Table 2:** CME Pre-test Post-test Results (N = 103)

Correct Response	Answered correctly on Pre-test n (%)	Answered correctly on Post-test n (%)	Δ	P-Value
Ductal carcinoma is the most commonly diagnosed breast cancer in women in the United States.	49 (47.5)	98 (95.1)	47.6%	.01

Sixty percent of breast cancers in women are diagnosed at a localized stage.	15 (14.5)	98 (95.1)	80.6%	.00

African Americans have the highest mortality rate from breast cancer in the United States.	83 (80.1)	64 (62.1)	-18.0%	.34

Recommendations from leading health organizations are in agreement that average risk women should be screened beginning at age 40.	24 (23.3)	59 (57.2)	33.9%	.09

Asian American/Pacific Islander's are least likely to participate in routine mammography screening.	15 (14.5)	98 (95.1)	80.6%	.00

The rate of mammography screening in the Unites States has decreased.	5 (4.8)	84 (82.0)	77.2%	.00

False-negative mammogram results are not a cause of breast cancer disparities.	54 (52.4)	88 (85.4)	33.0%	.06

Cultural beliefs can influence healthcare seeking.	98 (95.1)	98 (95.1)	0.0%	1.00

LEARN helps providers understand how provider bias influences medical recommendations.	15 (14.5)	83 (81.0)	66.5%	.00

The LEARN acronym stands for Listen, Explain, Acknowledge, Recommend, Negotiate.	74 (71.8)	93 (90.3)	19.0%	.22

Process evaluation data suggest that participants overall were well receptive to the CME course, content, and format (Table 3). Generally, participants responded very favorably that the learning objectives of the CME course were met. Similarly, the vast majority of participants agreed or strongly agreed that the content of the program was appropriate, easy to understand, and relevant to their practice. Ninety-five percent of participants agreed that the web based training was an appropriate tool to train health care providers about cultural competency. Further, 85% responded that web based course was easy to navigate.

**Table 3:** CME Process Evaluation (N = 103)

	Mean*	Agree		Strongly Agree		Agree and Strongly Agree Combined	
		n	%	n	%	n	%

**Learning Objectives**							

Discussed trends in Breast Cancer (BC) morbidity & mortality	4.54	41	40.0	59	57.3	100	97.3

Described evidence-based BC screening guidelines	4.62	39	38.0	64	62.0	103	100

Described factors that influence a patient's decision to undergo BC screening	4.65	36	35.0	67	65.0	103	100

Recognized how culture & cultural competence can influence BC screening	4.78	22	21.4	81	78.6	103	100

Described the LEARN model & understand its application to BC screening	4.79	21	20.0	82	80.0	103	100

**Course Content**							

The content was appropriate	4.65	36	35.0	67	65.0	103	100

The content was easy to understand	4.58	33	32.0	70	68.0	103	100

The content was relevant to the learning objectives	4.73	28	27.2	75	72.8	103	100

The content was relevant to your practice	4.46	43	41.7	54	52.4	97	94.1

The content facilitated learning	4.46	46	44.6	53	52.5	99	96.1

The web based training was appropriate	4.44	38	36.8	59	57.2	97	94.1

The web based course was easy to use/navigate	4.32	31	30.0	57	55.3	88	85.3

*Strongly Agree (5) - Strongly Disagree (1)

## Discussion

Although there are a host of factors that have been identified as potential causes of breast cancer disparities, the exact extent each plays in causing this disparity remains unknown. What is known is that early detection and screening is the best defense for reducing mortality seen for this cancer among underserved women. Yet underserved women are least likely to participate in mammography screening and, consequently, efforts are needed to ensure that these women understand the importance of early detection. An ideal environment for such effort is the clinic environment since it provides a unique opportunity to promote and counsel women about the benefits of early detection for breast cancer. However, data suggests that health care providers are not recommending preventive health services to their patients as recommended by the U.S. Preventive Services Task Force [30]. While the busy practice environment and time constraints account for some of the explanations of why women are not receiving mammography screening recommendations, there are other reasons. Based on formative work conducted in our needs assessment, we identified the inability of providers to effectively discuss breast cancer screening with their patients as a cause of low screening rates within the Montgomery Cares system. Evident was that providers had self conceived explanations and beliefs, with the most common being that women did not make time or did not have the resources to pay for screening, as reasons why their patients did not undergo or complete screening. In response, we created this CME course as means to update providers in primary care settings about breast cancer and screening recommendations and to also introduce a culturally appropriate framework that would give providers a tool to engage, discuss, and recommend breast cancer screening to women of diverse backgrounds.

Our CME course findings indicate that we were able to increase awareness about breast cancer disparities and a cultural competent communication framework. In examining pre- and post-test differences, we found that there were six learning outcomes that showed significant changes. Additionally, there was a significant increase in the number of individuals who correctly answered 70% of post-test questions. In all, we believe that we were able to increase CME participants' knowledge about breast cancer and the importance of cultural competence in the short term. Important to note is that some of the pre-test questions that deal with basic breast cancer facts and epidemiology were incorrectly answered at high rates and suggest that providers in general may need more information about breast cancer so that they can make better practice decisions.

One of the shortcomings of this CME, like others, is that the actual impact to healthcare provider practice behavior is not known. Future research needs to examine how courses that promote cultural competence and cross-cultural communication actually change practice behavior and lead to more meaningful physician-patient encounters. According to Campinha-Bacote [31], there is a direct relationship between providers' level of cultural competence and their ability to provider culturally appropriate services. In general, cultural competence is a promising approach to reducing health disparities, however more effort is needed that understands how interventions that attempt to change provider practice behavior ultimately reduce health disparities. Given that the Institute of Medicine has recommended cultural competency as a means for addressing and reducing health disparities [32], more research is warranted to better understand how health care delivered in a culturally competent manner influences patient health outcomes.

In developing this CME, we did not find any other CME or health care provider material that covered breast cancer screening and cultural competence together. Yet, surprisingly, breast cancer screening is perhaps an ideal health topic to teach the importance of cultural competence given the different beliefs and attitudes toward breast screening across cultural groups. Given that evidence clearly exists that suggests that cultural beliefs influence the decision to screen for breast cancer, this could be a good starting point for providers to understand the importance of cultural competence and its role when discussing breast cancer screening with patients. Further, using a cross-cultural communications for breast cancer screening could lead to adopting this practice behavior for all provider-patient interactions.

Overall, our CME course was well received and liked by participants as indicated by our process measures. In development, we explored other potential formats (i.e., in-person lecture, written material) that could be used. However, our formative research suggested that web-based was the preferred method of our target audience. Additionally, we chose an internet accessible platform since it allowed for easy access, flexibility, and could be easily updated for little additional cost. Further, we designed the web-based course to account for different learning styles by using both graphics and narration. Including vignettes and stories about patient interactions from actual healthcare providers also added to the CME and provided a multi-component learning environment. A review of internet and CD-Rom based continuing medical education (e-CE) suggests that e-CEs that are more interactive and multi-component in nature are more effective in changing knowledge and actual practice patterns of health care providers [33].

Although the purpose of this study was to develop and evaluate a CME course targeted to primary care providers, there are several limitations that should be acknowledged. First, we primarily focused on knowledge change and are not able to understand what impact this course had on physician practice patterns. A randomized design or a control group design was not used. The use of a single group pre- and post-test evaluation method may increase the likelihood of the Hawthorne effect due to asking knowledge questions at baseline [34], although some results went in the opposite direction of what would have been expected if there was a Hawthorne effect. Lastly, the course was open to all health professionals and convenience sampling was used. Participants who completed the CME were from the greater Washington DC metro area and limits study generalizability. In summary, our limitations are similar to other educational CMEs.

## Conclusions

Despite some of the limitations, our findings suggest that using a web-based CME course to educate healthcare providers about breast cancer disparities and the importance of cross-cultural communication was effective in changing intermediate outcomes. Additionally, the use of web-based CMEs may be a cost-effective strategy to train and educate healthcare providers about breast cancer disparities and cross-cultural communication. Given that only within the past decade have health professional school curricula been updated to teach cultural competency skills to students, there are numerous healthcare practitioners who have not received this training. The use of a web-based CME to educate providers about how cultural competence fits into the physician-patient interaction for discussing and recommending breast cancer screening presents a unique way to overcome this healthcare barrier and to possibly increase the use of mammography by underserved women. Healthcare providers who possess an understanding of the importance of culture in health care decision making and who are able to negotiate treatment plans that take into account a patient's cultural beliefs and practices will be able to breakdown the cultural discordance between provider and patient and could lead to increased trust and adherence with provider recommendations.

## Competing interests

The authors declare that they have no competing interests.

## Authors' contributions

RP conceptualized the study, participated in data collection, supervised data analysis, and drafted the manuscript. RS conceptualized the study, developed study materials, supervised data collection, and drafted the manuscript. MT conceptualized the study and interpretation of data. IM conceptualized the study, course materials, and interpretation of data. All authors helped review drafts of the manuscript and have read and approved the final manuscript.

## Pre-publication history

The pre-publication history for this paper can be accessed here:

http://www.biomedcentral.com/1472-6920/11/59/prepub
